# A Scoping Review of Military Culture, Military Identity, and Mental Health Outcomes in Military Personnel

**DOI:** 10.1093/milmed/usae276

**Published:** 2024-06-05

**Authors:** Carolyn Heward, Wendy Li, Ylona Chun Tie, Pippa Waterworth

**Affiliations:** James Cook University, Townsville, Queensland 4811, Australia; James Cook University, Townsville, Queensland 4811, Australia; James Cook University, Townsville, Queensland 4811, Australia; Independent Researcher

## Abstract

**Introduction:**

The military is a unique cultural institution that significantly influences its members, contributing to the development and transformation of their identities. Despite growing interest in identity research in the military, challenges persist in the conceptualization of military identity, including understanding how it forms, assessing the influence of military culture on identity development, and evaluating the implications for mental health. The primary objective of this scoping review was to map the complexities of military culture’s impact on military identity and its effects on mental health.

**Materials and Methods:**

A scoping review of the literature was conducted using the Joanna Briggs Institute Scoping Review Methodology. Studies were included if they described military culture, military identity, and mental health, resulting in 65 eligible studies. The extracted data were thematically analyzed to identify how military culture impacts military identity and mental health and well-being.

**Results:**

Multiple identities were evident within the military population, with 2 overarching identities, loyalty and military, overall conferring positive mental health outcomes. Where these identities were hidden or disrupted, poorer mental health outcomes were observed.

**Conclusions:**

The scoping review conducted in this study challenges the notion of military identity as a singular concept promoting positive mental health outcomes. It highlights its multifaceted nature, revealing that individuals may face identity concealment and disruptions during periods of transition or adjustment, resulting in adverse mental health outcomes. To capture the complexity of military identity, the authors developed the Military Identity Model (MIM). Military leaders, policymakers, and health care professionals are encouraged to recognize the complex nature of military identity and its impact on mental health and well-being. We recommend using the Military Identity Model to explore military identity and adjustment-related difficulties.

## INTRODUCTION

Active military personnel generally exhibit positive mental well-being, with mental health condition rates comparable to the general population, though with variability in presentation types.^1,2^ In the United States, 25.1% of military personnel met 30-day mental health disorder criteria, with 11.1% experiencing multiple conditions, including a higher prevalence of post-traumatic stress disorder (PTSD) than civilians.^1^ In Australia, ∼22% reported 12-month mental health disorders, with a greater prevalence of affective disorders and PTSD; a larger proportion met lifetime diagnostic criteria than nonmilitary personnel.^2^ In the United Kingdom, personnel with recent military operational experience displayed higher rates of common mental health conditions, alcohol use, and PTSD compared to civilians.^3^ These findings emphasize the need to explore how the unique cultural context of military life shapes mental health experiences within this population.

Military culture, a dynamic force shaped by shared beliefs, values, behaviors, norms, symbols, and practices, shapes individuals’ identities, worldviews, and social interactions.^4,5^ Through norms, traditions, rituals, values, lexicon, dress, training, and deployments, military culture governs interactions and fosters identity transformations among personnel.^6–9^ This deliberate shift from civilian to a military identity is crucial for adapting to military life.^10–12^

Military identity, a product of this unique cultural context, is further shaped and maintained by social processes,^13–17^ intertwines with personal identities, and provides a framework for positioning within the military sociocultural context.^18,19^ This identity formation process goes beyond simply understanding external characteristics like uniforms or hierarchies. It is an intricate psychological journey,^20,21^ where group membership permeates the self, shaping self-perception, norms, attitudes, emotions, beliefs, and behaviors.^22–26^ Challenges arise when norms, values, beliefs, attitudes, and behaviors of different identities conflict, potentially resulting in adverse mental health outcomes.^18,27,28^

Military service significantly impacts service members’ identities, shaping their self-perceptions, worldviews, and mental health and well-being. While some aspects of military identity are positive, like a strong centrality of veteran identity leading to psychological functioning^29,30^ and reduced suicidal thought,^31^ others are linked to negative consequences. Attachment aspects of identity, like interconnectedness and seeing the military-as-family, correlated with depression, PTSD, and negative affect.^29^ Furthermore, the military identity is focused on “doing,” which is the deeds and activities of soldiering,^32^ and this emphasis can adversely affect mental health when individuals face challenges in being unable to perform tasks, particularly during rehabilitation and military transition.^33^

The distress of conflicting combat soldier and civilian identities, often leading to moral injury,^34^ highlights the impact of identity strain on mental distress rather than trauma as its primary cause.^35^ Moral injury, recognized as a contributing factor to adverse mental health outcomes among military personnel,^36^ involves psychological distress stemming from actions or experiences that violate deeply held values.^37^ Conflicts between civilian and military values can trigger an identity crisis, eliciting self-oriented negative emotions such as guilt, shame, anxiety, and self-condemnation.^37^

While research has extensively examined the transition out of the military and resultant identity conflicts, revealing significant adverse effects on individuals’ mental health and suicide risk,^10,35,38–44^ limited research has explored how military service affects identity and mental health in the early years, aside from predicting failure in the military.^45–47^ A solitary study exploring mental health trends during the early years of military service identified a rise in negative mental health symptoms during the early years of military identity formation.^48^

Despite growing interest in military identity research, existing evidence primarily relies on quantitative measures and descriptive scales.^29,49,50^ Qualitative research has often relegated military identity to a subsidiary role by focusing on postdeployment reintegration^10,51^ and postdischarge adjustment.^38,39,52,53^ Scoping review by Dolan et al.^54^ emphasizes postdischarge salience neglecting the examination of military identity within active service. There is a lack of comprehensive exploration of military identity within active service, including the early years, its developmental trajectory, and its impact on mental health.^11,48,55^ This scoping review aims to address this gap by mapping the complexity of military culture, military identity, and their interplay with mental health by addressing the following research questions (RQs): (RQ1) What is military identity? (RQ2) What is the influence of military culture on military identity? (RQ3) What is the impact of military culture and military identity on military personnel’s mental health?

## METHODS

This scoping review followed Arksey and O’Malley’s^56^ framework with extensions by the Joanna Briggs Institute.^57,58^ Inclusion criteria encompassed empirical peer-reviewed articles, full conference papers, and theses/dissertations of various methodologies focusing on military personnel, military culture, and military identity or mental health. Exclusion criteria comprised non-English publications, nonmilitary subjects or child soldiers, and gray literature. Gray literature was not scoped because of its primarily governmental nature, lacking the military personnel’s perspective. A research librarian optimized the search strategy.

Eight databases (MEDLINE [OVID], PsychInfo [Proquest], Military Database [Proquest], PTSDpubs [Proquest], Cumulative Index to Nursing and Allied Health Literature, Emcare, Scopus, and PubMed) were searched from inception to July 2022. An updated search was performed in October 2023 to identify any additional relevant studies. The final search strategy for the MEDLINE database is presented in Supplementary Appendix 1.

Initial screening of titles and abstracts involved all authors. Full-text screening was conducted by C.H. with a subsequent review by W.L., disagreements in screening were resolved through discussion until consensus was reached. Data extraction was piloted on a subset of articles before full extraction commenced. Study characteristics extracted included author/s, year of publication, methodology, description of military culture and identity, the link between culture and identity, and reported mental health outcomes among military personnel.

Thematic analysis was employed for its ability to capture common themes across diverse study designs.^59^ The iterative review and coding of articles, guided by the RQs using an inductive approach, allowed for the identification of codes and themes directly from raw data without pre-existing frameworks. The final themes were derived from these codes. Article bias or quality appraisal was not considered.

## RESULTS

### Overview and Descriptive Summary

**FIGURE 1.**
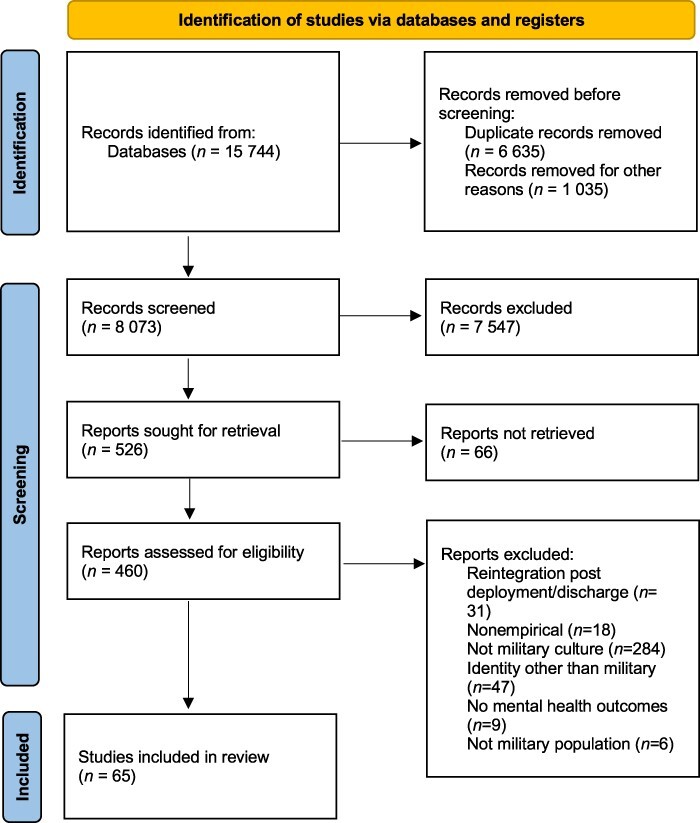
PRISMA flow diagram.

In total, 8073 titles and abstracts were reviewed with 7547 excluded; a further 66 could not be retrieved despite author contact. Following a rigorous review process, 65 original articles and dissertations (52 journal articles, 13 dissertations) were included in the final analysis (see Fig. 1 for the Preferred Reporting Items for Systematic Reviews and Meta-Analyses flowchart).

The majority of the research was conducted in the United States (74%), and most participants were male (82%). All military branches were included, with most reporting a combination of branches. The number of participants ranged from 3 to 4854, and ages ranged from 18 to 92 years. The studies included 23 quantitative, 38 qualitative, and 4 mixed-methods studies, with most published after 2015 (83% [54/65]). **Table I** summarizes data characteristics, and Supplementary Table S1 outlines extracted themes.

**TABLE I.** Data Characteristics Summary

Paper characteristic			
Type	Included papers (*n* = 65), *n*	Frequency (%)	
Quantitative research report	23	35.4	
Qualitative research report	38	58.5	
Mixed-methods research report	4	6.2	
Paper origin			
Australia	1	1.5	
Canada	4	6.2	
Germany	1	1.5	
Israel	4	6.2	
Norway	1	1.5	
Philippines	1	1.5	
Portugal	3	4.6	
United Kingdom	2	3.1	
United States	48	73.8	
Branch of service			
Air Force	1	1.5	
Army	10	15.4	
Marines	1	1.5	
Navy	1	1.5	
Not specifically stated/reported	19	29.2	
Mixed branches of service	33	50.8	
Publication year			
pre-2001	0	0.0	
2001 to 2010	3	4.6	
2011 to 2015	10	15.4	
2016 to 2020	29	44.6	
2021 to 2023	23	35.4	
Sex	Sample, *n*		
Male	14,485	69.6	81.8
Female	3230	15.5	18.2
Not reported	3101	14.9	
Sample not described (*n* studies)	1		
Age			
Range (years)	18 to 92		
Age not reported (*n* studies)	16		

### RQ1: Military Identities

The analysis identified varied military identities. The most prevalent identity identified were those associated with moral injury (46%), hidden identities (29%), and loss of identity (31%).

Morally injured identities (30/65 studies)^60–89^ were identified as a prominent theme, stemming from exposure to potentially morally injurious events (PMIEs) within the military context.^37^ The morally injured identity is characterized by feelings of guilt, violated expectations, and betrayals.

Hidden identities (19/65 studies) described the experience of suppressing or compartmentalizing emotions and true selves to conform to the perceived expectations of the military environment.^35,90–99^ Feminine conformity was detected as a subtheme within this category with 9 studies highlighting the challenges faced by women in adapting to masculine ideals and expectations within the military culture.^80,98,100–106^

Loss of identity (20/65 studies) was found to be a significant theme, particularly during the transition to civilian life postdeployment or discharge. Veterans described a sense of disconnection, confusion, and difficulty reintegrating into civilian society.^35,39,77,95,102,104,107^ This experience often involved feelings of not fitting in and questioning one’s sense of purpose and identity.^10,35,39,77,92,93,95,96,100,102,104,108–110^ Female veterans specifically reported feeling invisible and unrecognized as veterans in postdischarge life, as civilians did not perceive them as real veterans, perpetuating the notion that only men are veterans.^80,98,100,101,104^ Some participants felt discarded and conflicted about their roles, one starkly stated “I thought killing was my purpose.”^69 p. 402^ Notably, one study explored the loss of identity within current military service, focusing on medics revealing a loss of professional identity during deployment.^111^ After leaving the military, these medics saw themselves more positively, focusing on their helping roles rather than feeling futile.

Beyond these prevalent themes, other noteworthy identities were identified, including the loyal identity (18/65 studies) characterized by duty, comradeship, and immersion in the collective military group.^10,39,89,91,92,97,102,105–108,111–117^ The warrior identity (15/65 studies) was defined by notions of strength, invincibility, and lethality,^10,35,39,91,93,94,96,99–101,107,108,117–119^ and the stigmatized identity (5/65 studies) represents internalized stigma and was marked by feelings of weakness, silence, and instability.^90,95,110,120,121^ Notably, this identity is accompanied by an acknowledged ‘catch-22’ whereby “if you know you need help than (sic) you are rational; but if you actually seek help, then you are crazy and not trustworthy to do your job.”^,110 p. 4288^

Additionally, 3 studies discussed the failed warrior identity, whose failing led to the development of combat-related PTSD and a “fall from the masculine grace.”^,99,118,119 p. 217^ Two studies described the spoiled warrior identity^35,100^ as individuals who had not deployed and described themselves as “feel(ing) an unfulfilled void that plagues their sense of self—a kind of “‘spoiled identity.’”^35 p. 153^

### RQ2: Military Culture Link to Military Identity

A strong link was established between military culture and military identity, with military culture shaping both favorable and disrupted identities. The voluntary adoption of group norms highlighted in 12 publications played a significant role in shaping military identity.^10,39,89,97,103,108,109,112,113,116–118^ Participants described learning the rules of the game,^10^ forging loyalty,^39,117^ and prioritizing group norms over individual beliefs being central to military identity.^39^

The pressure to conform to military culture was identified as a significant theme, with 15 studies exploring the challenges faced by individuals in navigating these expectations.^35,90,99,100,102,104,110,114,115,119–121^ Women specifically faced unique pressures to distance themselves from their femininity,^106^ play a character to fit in,^98^ and navigate the inclusivity of the military institution, conditional on “fit(ting) the stereotypical notion of what a service member is supposed to be like and behave—a hypermasculine, ‘warrior identity’.”^105 p. 9^

Military training was a key factor, identified in 7 studies, in shaping both expectations and group norms within the military context, ultimately influencing individual identity.^10,92–94,96,101,107^ These studies emphasized how military training breaks down and rebuilds individuals into warriors,^93,101^ not as an innate trait but a result of training.^96^ Four studies reported that military culture influenced identity through established rituals and routines.^91,95,109,111^ These practices provided a structured framework and reinforced military identity by incorporating symbolic elements that reflect the core values and purpose of the military.^91^

The most prominent theme exploring the complex link between military culture and military identity arose from moral conflicts (30/65 studies).^60–89^ These conflicts stemmed from situations where individuals were expected to conform to the military culture and to act in ways that contradicted their deeply held beliefs or witnessed moral transgressions committed by others. Betrayal, a subtheme in 14 of the aforementioned 30 publications, reflected disappointment and let down in the perception of what military culture should provide.^60–63,66,68–71,80,81,83,85,87^ Instances of betrayal were evident in reports of leadership failures, where commanders indifferent to the survival or death of their subordinates^66^ impacts the individual’s identity as “their core belief that the military takes care of them has been violated.”^68 p. NP10012^ Women in the military also faced the impact of betrayal, as they experienced belittlement based on traditionally associated feminine personality traits.^80^ These experiences often led to a dissonance between the individual’s personal moral compass and the perceived expectations of the military culture, ultimately impacting their sense of self and potentially contributing to the development of a morally injured identity.^65,66^

### RQ3: Mental Health in Military Personnel

The impact of military culture and military identity on mental health was found to be complex and multifaceted. The formation of favorable or positive identities (e.g., loyal, warrior), particularly when individuals felt they could express these identities authentically without the need to hide or experience disruption, was associated with positive mental health outcomes (11 studies). These outcomes included feelings of pride,^39,97,113^ belonging, and overall positive adjustments, like feeling confident, capable, and resilient.^39,91,93,101,105,107,112,113,117^

Conversely, 3 studies reported negative mental health outcomes in relation to fitting into the loyal/warrior identity, risky or hazardous alcohol use,^114,116^ and the negative aspects of fitting in on self-esteem because of weight scrutiny.^115^

The need to hide or suppress elements of identity to fit in resulted in a range of negative mental health outcomes. A total of 15 studies discussed negative outcomes, with emotional suppression being the most common (10 studies)^35,91–94,96–99,103^ and with one study describing military personnel as “lacking a language of distress.”^,93 p. 1484^ Anger in response to hiding elements of identity was described in 2 studies.^91,101^ Women in hiding their femininity reported feeling harassed and threatened,^102^ betrayed,^100^ and excluded.^104^ Additionally, women described the negative consequence of role conflict^97,101^ on self-esteem^105^ and the internalization of inferiority.^106^ Two articles described the use of substances to cope with the impact,^93,94^ whereas positive mental health outcomes of resilience, optimism, and pride, despite hiding their femininity, were highlighted in 3 studies.^80,103,104^

Disruptions to loyal and warrior identities that were perceived to have failed often led to feelings of guilt, shame, emasculation,^118,119^ and perceiving oneself as weak.^99^ Similarly disruptions because of self-stigma damaged mental health, causing problematic alcohol use,^120^ guilt,^121^ shame and weakness,^90^ and feeling dehumanized, increasing suicide risk.^110^ The spoiled identity, stemming from not having deployed, resulted in lowered self-esteem.^35,100^ The disrupted loss of identity because of reintegration struggles resulted in distress and adverse mental health impacts such as anger or destructive behaviors,^35,96,107,109^ alcohol use,^69^ disconnection and poor life satisfaction,^39,92,100,104,108,109^ and suicidal ideation.^95,96^ Identity crises, especially of feeling senseless and lacking purpose, were reported most frequently for the lost identity and were linked to lost self-worth.^10,69,101,102,110,111^

The morally injured identity, predominantly exhibiting symptoms related to moral injury, such as shame, guilt, self-stigma, poor self-compassion, struggles with meaning making and forgiveness (14/65 studies).^61,66,67,69,72,73,76,77,81–83,86,87,89^ Additionally, common mental health disorders were described in 11 studies^60,63,65,68,72–74,79,80,82,83^ along with PTSD and trauma-related symptoms (10 studies),^63,65,67,70,72,74,75,80,82^ suicidal ideation and behavior (11 studies),^62,67,68,71–74,79–82^ and problematic substance use (11 studies).^60,61,69,73,75,77,78,81,82,84,87^ Other adverse mental health impacts were noted in 11 studies and included anger, compartmentalization of emotions, negative cognitions, self-esteem impacts, withdrawal, and interpersonal difficulties.^61,70,71,73,74,76,80,81,84,85,88^

While the majority of studies focused on the negative mental health consequences associated with moral injury, 5 studies examined positive outcomes, primarily focusing on post-traumatic growth.^64,77,86^ One publication outlined the role a sense of purpose in life plays in mitigating suicide risk following transgressive experiences by others,^62^ whereas another highlighted the involvement in prosocial actions as ways for individuals to find meaning and compensate for past moral transgressions.^69^

## DISCUSSION

This scoping review examined 65 studies exploring military identity, its connection to military culture, and its impact on mental health. Guided by the RQs, the review provided an extensive analysis of military identities, the cultural influences shaping them, and subsequent mental health outcomes.

### Understanding Military Identities

The review revealed the multifaceted nature of military identities with several prevalent forms and subcategories. Among these, the loyal and warrior identities were identified as aligning most closely with the military culture’s norms, traditions, and values. These often coexisted within individuals, with numerous studies noting this phenomenon.^10,39,91,107,117^ However, these identities often masked a hidden/invisible subidentity adopted in order to fit it. More generally, this required the hiding of the emotional self and, for women, conforming to masculine ideals. This aligns with the emphasis on stoicism and suppression vulnerabilities, fostered from recruit training onward, enhancing short-term adaptability and mission success and prevailing throughout an individual’s career.^122^

Subordinate to the loyal and warrior identities, the review identified disrupted identities influenced by various factors. The morally injured identity, arising from exposure to PMIEs, was the most frequently disrupted identity and likely represents an increased interest in the consequences of exposure to PMIEs. The failed warrior identity developed when individuals perceived that their failing to uphold masculine ideals led to the development of combat-related PTSD. The stigmatized identity was characterized by internalizing stigma and viewing ones’ self as being weak, silent, and unstable. The stigmatized identity is likely related to the failed warrior identity in that stigma is a component of each; however, the failed warrior subcategory differs in that there is specific articulation of the failure to uphold the warrior strength. The review identified a subgroup within the warrior identity, those who hadn’t deployed. This group labelled spoiled warriors, described themselves as spoiled due to feeling unfulfilled as a warrior, impacting their sense of self.

Lastly, the loss of identity represents a significant disrupted identity, often related to the challenges of transitioning to civilian life after deployment or discharge from the military. This is aligned with the concept of diminished self or identity proposed by Brewin, Garnett, and Andrews^123^ in that individuals described feeling amnesic, disconnected, confused, and struggling to fit into civilian society. Female veterans reported feeling invisible, and some participants expressed feelings of being discarded and conflicted, further highlighting the complexity of this identity. This scoping review provides valuable insights into the complexity and inter-relatedness of identities within the military population, contributing to a deeper understanding of the experiences of military personnel.

### Military Culture and Military Identity Formation

The review highlighted how conditioning and social learning through training, adaptation, and voluntary alignment with group norms and rituals shape identity formation. This often involves prioritizing collective interests and silencing personal aspects. These findings concur with the tenets of social identity theory, emphasizing the significance of the group and social context in shaping one’s identity.^6,7,18,19,23,26^

Furthermore, the review highlights the influence of moral conflicts on military identity. The morally injured identity arises from the dissonance between military and civilian rules, highlighting how military service ethos and combat exposure might influence identity formation; however, the precise relationship between potential moral conflict arising from these factors and its association with military culture remains the subject of ongoing investigation, with ongoing efforts to strengthen our understanding.^37,124,125^

Overall, the review underscores the multifaceted and dynamic interplay between military culture and identity. With identities evolving over time and influenced by various factors and events, understanding these influences is vital for supporting military personnel as they navigate the complexities of their identities and experiences in their service journey.

### Impact on Mental Health

The findings revealed a spectrum of outcomes, with positive mental health outcomes associated with the loyal and warrior identities and a greater prevalence of negative mental health when identities are hidden or disrupted. The emphasis on self-reliance and stoicism within the military can create barriers for help-seeking for veterans facing mental health challenges. This aligns with the existing literature exploring the potential negative consequences of stoicism and traditional masculinity, manifesting as impaired psychological adjustment, perfectionism, and limitations on overall well-being.^126,127^ Additionally, the stigma and culture of silence surrounding mental health further hinder help-seeking. Addressing these detrimental effects is crucial for promoting mental health equity and fostering a more supportive environment.^128–130^

Importantly, the review countered the prevalent focus on negative mental health outcomes by illuminating the often-overlooked positive aspects fostered by military culture and identity. The sense of pride, purpose, and belonging cultivated within the military serves as a source of support and resilience to navigate the stressors inherent in service. These positive aspects contribute to overall well-being, aligning with the acknowledgment of the military as a positive institution dedicated to personnel welfare.^131^ However, further exploration of these positive dimensions is warranted.

### The Military Identity Model

**FIGURE 2.**
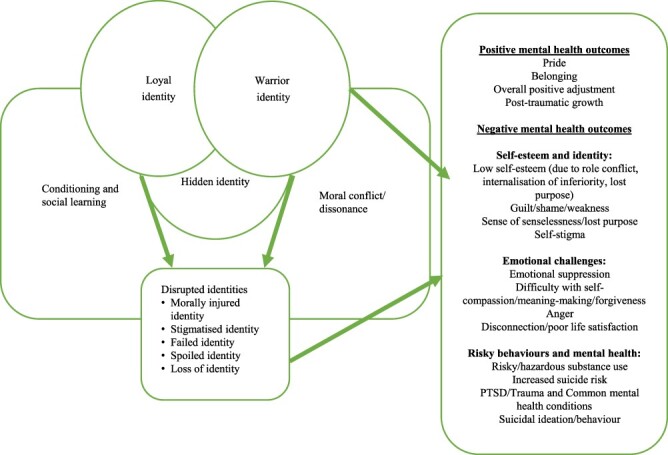
Military Identity Model.

^a^Reproduced with permission from C.H.^140^. The image of the Military Identity Model produced in the manuscript was created by the first author, C.H., written permission for its use follows. I, C.H., hereby grant Military Medicine permission to use the image titled Military Identity Model, which I created and hold the copyright for, in the publication of my manuscript titled “A Scoping Review of Military Culture, Military Identity and Mental Health Outcomes in Military Personnel.” This permission is granted under the terms of the Creative Commons Attribution 4.0 International License (CC BY 4.0).

Drawing on the review’s findings, a model of military identity (Military Identity Model [MIM]) was developed (Fig. 2). Recognizing the presence of multiple, rather than singular, military identities, the MIM illustrates the intricate interplay between military culture and identity through conditioning, social learning, and dissonance and the resultant impact on mental health.

### Limitations

This scoping review on military culture, identities, and mental health, while comprehensive, has limitations. It excludes studies that utilized veteran populations as convenience samples. Although these studies explored physical or mental health conditions, replacing the military sample with a nonmilitary sample would have achieved equivalent research goals. This observation aligns with a recent systematic review of veteran’s studies.^55^ A further limitation is the exclusion of studies not explicitly discussing military culture. Articles on deployment’s impact on identity/mental health without explicit reference to military culture were excluded. Studies on other identities (e.g., Lesbian, Gay, Bisexual, Transgender, Intersex and Queer (LGBTIQ)+, ethnoracial)^132,133–139^ were also excluded. While acknowledging LGBTIQ+ identities may be concealed and ethnoracial minorities may repress the salience of their ethnoracial identity, this scoping review cannot draw definitive conclusions in these areas because of their exclusion.

The review’s scope was further constrained by its restriction to studies published in English, potentially overlooking valuable insights from non-English-speaking military research. The predominant focus on Western first-world nations, particularly the United States, raises questions about generalizability to different global contexts especially given the heterogeneity of militaries worldwide. The absence of articles older than 2007 and a historical dearth of military identity research suggest the need for further exploration, a trend also noted by Lira and Chandrasekar^55^ and Thompson et al.^11^ This scoping review, despite limitations, provides valuable insights into the complexities of military identity and its association with military culture and mental health, offering implications for practice and research in this field.

### Implications

The scoping review’s findings and the proposed MIM have significant implications for military policy, health care practice, and research. Military leaders and policymakers can gain insights from the MIM to navigate the intricacies of military identities, guiding the development of strategies to safeguard against compartmentalization and identity disruption. Likewise, health care professionals can utilize the MIM to understand the complex aspects of military identities. The model cautions against presuming a uniform identity among military personnel, encouraging health care professionals to explore individual experiences with military culture, identity, and mental health. Prioritizing identity in their work with military personnel can enhance their ability to provide tailored and effective health care.

This scoping review to the authors’ knowledge represents the first comprehensive synthesis of military identity and its relationship to military culture and mental health. While providing valuable insights, it also identifies limitations in explaining identity development. The review primarily emphasizes themes related to behavioral models of conditioning and social learning and cognitive models of dissonance, yet a gap exists in understanding the civilian-to-military identity transformation process. Additionally, longitudinal studies tracking identity evolution throughout the military journey could offer valuable insights into its dynamic nature and how various stages of service influence it. Future research endeavors in this direction are recommended.

## CONCLUSION

This scoping review has laid a foundation for understanding military identities and their interplay with military culture and mental health. The findings indicate that military identities can contribute positively to mental health outcomes, fostering belonging, pride, and resilience. However, the hiding or disruption of subidentities and the lack of acknowledgment of diverse identities can have negative mental health outcomes.

By illuminating the complexities of military identity and its implications, this scoping review lays the foundation for future investigations and underscores the urgent need to prioritize the exploration of military identity for the development of effective support systems and interventions that enhance the well-being of military personnel. The MIM offers guidance for military leaders, policymakers, and health care professionals in comprehending the multifaceted nature of military identities in order to support the mental health of those who serve.

## Supplementary Material

usae276_Supp

## Data Availability

Data not incorporated in this article and its online supplementary material are available upon reasonable request to the corresponding author.
