# Supplementary material for: A Scoping Review of Military Culture, Military Identity, and Mental Health Outcomes in Military Personnel
Source: Mil Med. 2024 Jun 5;189(11-12):e2382–93. doi: 10.1093/milmed/usae276 (PMC11536329; doi:10.1093/milmed/usae276)
Supplement: usae276_Supp [file usae276_supp.zip › Military Culture, Identity & Mental Health_Supplemental.docx]

**Supplementary Appendix 1: MEDLINE search strategy**

| **#** | **Query** |
| --- | --- |
| 1 | Military Personnel/ |
| 2 | Military Health/ |
| 3 | Veterans/ |
| 4 | (military or "armed forces" or "armed services" or "defen?e force defen?e personnel" or "defen?e services" or army or navy or airforce or "air-force" or RAAF or "special forces" or "defen?e member*" or servicem?n or servicewom?n or "active duty personnel" or "coast guard*" or soldier* or submariner* or veteran* or commando* or "green beret*" or reservist* or infantry or cadet).mp. [mp=title, abstract, original title, name of substance word, subject heading word, floating sub-heading word, keyword heading word, organism supplementary concept word, protocol supplementary concept word, rare disease supplementary concept word, unique identifier, synonyms] |
| 5 | 1 or 2 or 3 or 4 |
| 6 | Culture/ |
| 7 | Acculturation/ |
| 8 | Cultural Characteristics/ |
| 9 | Cultural Diversity/ |
| 10 | Organizational Culture/ |
| 11 | (cultur* or customs or lifestyle* or norm* or tradition* or value* or ritual* or convention* or belief* or ethnology or "way* of life" or acculturat* or assimiliat* or integrat*).mp. [mp=title, abstract, original title, name of substance word, subject heading word, floating sub-heading word, keyword heading word, organism supplementary concept word, protocol supplementary concept word, rare disease supplementary concept word, unique identifier, synonyms] |
| 12 | 6 or 7 or 8 or 9 or 10 or 11 |
| 13 | Identification, Psychological/ |
| 14 | Personality/ |
| 15 | Human Characteristics/ |
| 16 | Character/ |
| 17 | Individuality/ |
| 18 | Self Concept/ |
| 19 | (identit* or self* or selves or uniqueness or character or individualit* or personalit* or personhood).mp. [mp=title, abstract, original title, name of substance word, subject heading word, floating sub-heading word, keyword heading word, organism supplementary concept word, protocol supplementary concept word, rare disease supplementary concept word, unique identifier, synonyms] |
| 20 | 13 or 14 or 15 or 16 or 17 or 18 or 19 |
| 21 | Mental Health/ |
| 22 | Mental Disorders/ |
| 23 | exp Anxiety Disorders/ |
| 24 | exp Mood Disorders/ |
| 25 | exp Substance-Related Disorders/ |
| 26 | exp "Trauma and Stressor Related Disorders"/ |
| 27 | Occupational Stress/ |
| 28 | Stress, Psychological/ |
| 29 | "Quality of Life"/ |
| 30 | ("mental health" or "mental disorder*" or "mental illness" or "mental disease" or "mental ill health" or "mental distress" or "psychological illness" or "psychological distress" or "psychological disorder" or "psychiatric illness" or "psychiatric distress" or "psychiatric disorder" or "emotional disorder" or "emotional instability" or "emotional distress" or maladjustment or "behavio?ral symptom*" or "emotional regulat*" or distress or suicid* or "self-harm" or gambling or "substance abuse" or addiction* or alcoholi* or "drug abuse" or "affective disorder" or "mood disorder" or depressi* or anxi* or "adjustment disorder" or "sleep disorder" or "posttraumatic stress" or "post-traumatic stress" or "post traumatic stress" or PTSD or "moral injury" or "psychological well*" or wellbeing or "well-being" or "life satisfaction" or QoL or "quality of life" or resilience).mp. [mp=title, abstract, original title, name of substance word, subject heading word, floating sub-heading word, keyword heading word, organism supplementary concept word, protocol supplementary concept word, rare disease supplementary concept word, unique identifier, synonyms] |
| 31 | 21 or 22 or 23 or 24 or 25 or 26 or 27 or 28 or 29 or 30 |
| 32 | 5 and 12 and 20 and 31 |

**Supplementary Table S1**

*Included Studies and their Key Findings*

| **Evidence source Details and Characteristics** | **Findings on military identity, military culture and mental health outcomes** | | |
| --- | --- | --- | --- |
| Citation details | Identity Themes | Culture link to identity | Mental Health Outcomes |
| Abraham et al., 2018 | Loyal Warrior | Voluntary adoption of group norms | Belonging, positive adjustments (confident, capable, resilient) |
| Ahlfs, 2019 | Loyal Warrior Identity Loss | Military training | Belonging, positive adjustments (confident, capable, resilient) Distress and tension in reintegration |
| Ames et al., 2007 | Loyal | Pressure to conform | Risky or hazardous alcohol use |
| Ashwal-Malka et al., 2022 | Morally injured | Moral conflict/dissonance | CMD Substance use |
| Azevedo & Pereira, 2023 | Loyal Hidden | Pressure to conform | Belonging Self-esteem (role conflict/internalisation of inferiority) |
| Barnett et al., 2021 | Loyal Warrior Identity Loss | Voluntary adoption of group norms | Pride Belonging Disconnected/poor life satisfaction |
| Bauman, 2009 | Loyal Hidden Identity Loss | Military training | Emotional suppression Disconnected/poor life satisfaction |
| Berezin Cohen & Netzer, 2023 | Loyal Hidden | Pressure to conform | Self-esteem (role conflict/internalisation of inferiority) |
| Bobek, 2013 | Morally injured | Moral conflict/dissonance | Shame/guilt/self-stigma/poor self-compassion/poor meaning/poor forgiveness Substance use Adverse mental health impacts (i.e., anger/negative cognitions/self-esteem) |
| Boscarino, et al., 2022 | Morally injured | Moral conflict/dissonance | Substance use Adverse mental health impacts (i.e., anger/negative cognitions/self-esteem) |
| Breland et al., 2017 | Loyal | Pressure to conform | Self-esteem (weight-related) |
| Britt et al., 2015 | Stigmatised | Pressure to conform | Risky/hazardous alcohol use |
| Buelna, 2017 | Stigmatised | Pressure to conform | Guilt/shame/weakness |
| Corona et al., 2019 | Morally injured | Moral conflict/dissonance | Suicidal ideation/behaviour Sense of purpose --> mitigates suicide risk |
| De Guzman et al., 2017 | Warrior Failed | Voluntary adoption of group norms | Guilt/shame/emasculation |
| de Medeiros & Rubinstein, 2016 | Loyal Warrior Hidden | Rituals and routines | Belonging Emotional Suppression Anger |
| de Rond & Lok, 2016 | Loyal Identity Loss | Rituals and routines | Senseless/Lost purpose --> lost self esteem |
| Demers, 2011 | Loyal Warrior Identity Loss | Voluntary adoption of group norms Military training | Senseless/Lost purpose --> lost self esteem |
| Demers, 2013 | Loyal Hidden Identity Loss | Pressure to conform | Harrassed/threatened Senseless/Lost purpose --> lost self esteem |
| Denneson et al., 2015 | Hidden Stigmatised Identity Loss | Rituals and routines | Suicidal ideation |
| DiNatale, 2021 | Warrior Hidden Identity Loss | Military training | Emotional suppression Suicidal ideation Adverse mental health impacts (i.e., anger/destructive behaviours) |
| Eichler, 2022 | Warrior Hidden Spoiled Identity Loss | Pressure to conform | Betrayed Disconnected/poor life satisfaction |
| Eskenasi, 2020 | Warrior Hidden Identity Loss | Military training | Belonging Self-esteem (role conflict/internalisation of inferiority) Anger Senseless/Lost purpose --> lost self esteem |
| Evans, Stanley et al., 2018 | Morally injured | Moral conflict/dissonance | CMD PTSD/Trauma |
| Evans, Szabo et al., 2018 | Morally injured | Moral conflict/dissonance | Post traumatic growth |
| Ferrajao & Oliveira, 2014 | Morally injured | Moral conflict/dissonance | CMD PTSD/Trauma |
| Ferrajao & Oliveira, 2015 | Morally injured | Moral conflict/dissonance | Shame/guilt/self-stigma/poor self-compassion/poor meaning/poor forgiveness |
| Frazier et al., 2017 | Morally injured | Moral conflict/dissonance | Shame/guilt/self-stigma/poor self-compassion/poor meaning/poor forgiveness PTSD/Trauma Suicidal ideation/behaviour |
| Green et al., 2010 | Warrior Hidden Identity Loss | Military training | Belonging  Emotional suppression Substance use |
| Hamrick et al., 2022 | Morally injured | Moral conflict/dissonance | CMD Suicidal ideation/behaviour |
| Hansen, 2023 | Morally injured | Moral conflict/dissonance | Adverse mental health impacts (i.e., anger/negative cognitions/self-esteem) |
| Hardy, 2023 | Hidden Identity Loss | Pressure to conform | Emotional suppression |
| Held et al., 2017 | Morally injured | Moral conflict/dissonance | PTSD/Trauma Adverse mental health impacts (i.e., anger/negative cognitions/self-esteem) |
| Held et al., 2018 | Morally injured Identity Loss | Moral conflict/dissonance | Shame/guilt/self-stigma/poor self-compassion/poor meaning/poor forgiveness Substance use Prosocial actions --> finding meaning/compensation |
| Houtsma et al., 2017 | Morally injured | Moral conflict/dissonance | Suicidal ideation/behaviour Adverse mental health impacts (i.e., anger/negative cognitions/self-esteem) |
| Jones et al., 2012 | Loyal | Voluntary adoption of group norms | Belonging, positive adjustments (confident, capable, resilient) |
| Kalmbach, et al., 2023 | Morally injured | Moral conflict/dissonance | Shame/guilt/self-stigma/poor self-compassion/poor meaning/poor forgiveness Post traumatic growth |
| Kaspersen, 2023 | Loyal Morally injured | Voluntary adoption of group norms Moral conflict/dissonance | Shame/guilt/self-stigma/poor self-compassion/poor meaning/poor forgiveness |
| Kelley et al., 2021 | Morally injured | Moral conflict/dissonance | PTSD/Trauma Substance use |
| Kelley, Braitman et al., 2019 | Morally injured | Moral conflict/dissonance | Shame/guilt/self-stigma/poor self-compassion/poor meaning/poor forgiveness CMD PTSD/Trauma Suicidal ideation/behaviour |
| Kelley, Bravo, Davies, et al., 2019 | Morally injured | Moral conflict/dissonance | Shame/guilt/self-stigma/poor self-compassion/poor meaning/poor forgiveness CMD PTSD/Trauma Suicidal ideation/behaviour Substance use Adverse mental health impacts (i.e., anger/negative cognitions/self-esteem) |
| Kelley, Bravo, Hamrick et al., 2019 | Morally injured | Moral conflict/dissonance | CMD PTSD/Trauma Suicidal ideation/behaviour Adverse mental health impacts (i.e., anger/negative cognitions/self-esteem) |
| LeFeber & Solorzano, 2019 | Stigmatised  Identity Loss | Pressure to conform | Increased suicide risk Senseless/Lost purpose --> lost self esteem |
| Maguen, et al., 2023 | Morally injured | Moral conflict/dissonance | Shame/guilt/self-stigma/poor self-compassion/poor meaning/poor forgiveness Substance use |
| Mayer, et al., 2023 | Identity Loss | Voluntary adoption of group norms Ritual and rules | Adverse mental health impacts (i.e., anger/destructive behaviours) Senseless/Lost purpose --> lost self esteem |
| McCarthy, 2017 | Morally injured | Moral conflict/dissonance | Shame/guilt/self-stigma/poor self-compassion/poor meaning/poor forgiveness Adverse mental health impacts (i.e., anger/negative cognitions/self-esteem) |
| McCaslin et al., 2021 | Loyal Hidden | Voluntary adoption of group norms | Pride Emotional suppression Self-esteem (role conflict/internalisation of inferiority) |
| McCormack et al., 2021 | Morally injured Identity Loss | Moral conflict/dissonance | Shame/guilt/self-stigma/poor self-compassion/poor meaning/poor forgiveness Substance use Post traumatic growth |
| Peppard et al., 2022 | Hidden | Voluntary adoption of group norms | Emotional suppression Resilience/optimism/pride in achievement |
| Robbins, 2016 | Morally injured | Moral conflict/dissonance | Substance use |
| Rotter, 2016 | Loyal | Voluntary adoption of group norms | Pride Belonging, positive adjustments (confident, capable, resilient) |
| Rusch et al., 2017 | Hidden Stigmatised | Pressure to conform | Guilt/shame/weakness |
| Ryu, 2023 | Morally injured | Moral conflict/dissonance | Adverse mental health impacts (i.e., anger/negative cognitions/self-esteem) |
| Saunders et al., 2021 | Hidden Identity Loss | Pressure to conform | Excluded Disconnection/poor life satisfaction Resilience/optimism/pride |
| Schumacher, 2018 | Morally injured | Moral conflict/dissonance | CMD Suicidal ideation/behaviour |
| Senecal et al., 2019 | Loyal Warrior Identity Loss | Voluntary adoption of group norms | Disconnection/poor life satisfaction |
| Shields et al., 2017 | Warrior Failed | Pressure to conform | Guilt/shame/emasculation |
| Silvestrini & Chen, 2023 | Warrior Hidden Failed | Pressure to conform | Emotional suppression Weakness |
| Smith & True, 2014 | Warrior Hidden Spoiled Identity Loss | Pressure to conform | Emotional suppression Poor self-esteem Adverse mental health impacts (i.e., anger/destructive behaviours) |
| Steeves, 2020 | Morally injured Hidden Identity Loss | Moral conflict/dissonance | CMD PTSD/Trauma Suicidal ideation/behaviour Adverse mental health impacts (i.e., anger/negative cognitions/self-esteem) Resilience/optimism/pride |
| Sullivan & Starnino, 2019 | Morally injured | Moral conflict/dissonance | Shame/guilt/self-stigma/poor self-compassion/poor meaning/poor forgiveness Suicidal ideation/behaviour Substance use Adverse mental health impacts (i.e., anger/negative cognitions/self-esteem) |
| True et al., 2015 | Warrior Hidden | Military training | Emotional suppression Substance use |
| Williams & Berenbaum, 2019 | Morally injured | Moral conflict/dissonance | Shame/guilt/self-stigma/poor self-compassion/poor meaning/poor forgiveness PTSD/Trauma Suicidal ideation/behaviour Substance use |
| Woodruff et al., 2018 | Loyal | Voluntary adoption of group norms | Risky/hazardous alcohol use |
| Zerach et al., 2021 | Morally injured | Moral conflict/dissonance | Shame/guilt/self-stigma/poor self-compassion/poor meaning/poor forgiveness CMD |

*Note*: CMD: Common Mental Health Disorder PTSD: Post-traumatic stress disorder
